# Elevated C‐X‐C motif ligand 13 and B‐cell‐activating factor levels in neuromyelitis optica during remission

**DOI:** 10.1002/brb3.648

**Published:** 2017-03-10

**Authors:** Su Wang, Tao Yang, Jianglong Wan, Yongchao Zhang, Yongping Fan

**Affiliations:** ^1^ Department of Traditional Chinese MedicineBeijing Tiantan HospitalCapital Medical UniversityBeijingChina; ^2^ Department of OncologyQingdao Hiser Medical GroupQingdaoChina; ^3^ Department of Traditional Chinese MedicineMiyun Xitiangezhuang Town Community Health Service CenterBeijingChina; ^4^ Department of Traumatic OrthopedicsQingdao Hiser Medical GroupQingdaoChina

**Keywords:** B‐cell‐activating factor, B cells, C‐X‐C motif ligand 13, immunosuppressive therapy, neuromyelitis optica

## Abstract

**Background:**

Discovery of specific antibodies against astrocytic water channel aquaporin‐4 (AQP4), which is produced by plasma cells, in the serum of neuromyelitis optica (NMO) confirmed the pathogenic role of B cells in NMO. C‐X‐C motif ligand 13 (CXCL13) and B‐cell‐activating factor (BAFF) are two crucial factors for antibody production. Relevant studies have focused on the acute phase of NMO. However, CXCL13 and BAFF levels during remission, remain to be elucidated.

**Objective:**

To evaluate serum levels of CXCL13 and BAFF in NMO and multiple sclerosis (MS) patients during remission and explore their correlation with immunosuppressive agents and clinical features in NMO.

**Methods:**

Serum CXCL13 and BAFF were measured by enzyme‐linked immunosorbent assay (ELISA) in NMO patients, MS patients, and controls.

**Results:**

Serum CXCL13 levels of NMO patients (*n *=* *24) were significantly higher than those of controls (*n *=* *22) (*p *=* *.001), but CXCL13 levels of MS patients (*n *=* *20) and controls (*n *=* *22) did not differ significantly (*p *=* *.279). Although the three groups showed no differences in serum BAFF levels, serum BAFF levels of NMO patients without immunosuppressive treatment (*n *=* *8) were significantly elevated compared with those of NMO patients with immunosuppressive therapy (*n *=* *16) (*p *=* *.003) and controls (*n *=* *22) (*p *=* *.024). In NMO patients, CXCL13 levels were correlated with onset age (*p *=* *.026) and duration to the last relapse (*p *=* *.003).

**Conclusion:**

During remission, serum CXCL13 and BAFF levels have not decreased to normal in NMO patients, and B‐cell‐related autoimmune response persists. Immunosuppressive therapy decreased serum BAFF levels, but did not affect CXCL13 expression.

## 1 Introduction

Neuromyelitis optica (NMO), an inflammatory demyelinating disease of the central nervous system (CNS) that most commonly affects young adults, has been considered a subtype of multiple sclerosis (MS) for many years (Carlsen, Baekkevold, Morton, Haraldsen, & Brandtzaeg, 2004; Gunn et al., 1998). Accumulating pathological, therapeutic, and experimental evidence indicates that B cells play a pathogenic role in both NMO and MS (Krumbholz & Meinl, 2014), especially the former. Studies have found immunoglobulin deposition in active lesions of the two diseases (Lucchinetti et al., 2000, 2002), as well as reduction in NMO relapses with treatment of selective B‐cell depletion by rituximab (Pellkofer et al., 2011). The demonstration of AQP4‐specific immunoglobulin G (IgG) autoantibodies (NMO‐IgG) in NMO patients (Lennon, Kryzer, Pittock, Verkman, & Hinson, 2005; Lennon et al., 2004), and its pathogenicity (Saadoun et al., 2010; Tradtrantip et al., 2012) provides further evidence. C‐X‐C motif ligand 13 (CXCL13) and B‐cell‐activating factor (BAFF) are two decisive factors in B‐cell migration (Gunn et al., 1998), survival, and maturation (Batten et al., 2000) and both are essential for immune function.

C‐X‐C motif ligand 13 is currently considered a potential biomarker of NMO. Serum and cerebrospinal fluid (CSF) concentrations of CXCL13 are reportedly elevated in NMO patients during relapse; furthermore, CXCL13 levels have a higher trend in NMO than in MS (Alvarez et al., 2013; Festa et al., 2009; Quan et al., 2013; Zhong et al., 2011). Meanwhile, studies have demonstrated that CXCL13 levels have a strong correlation with some clinical measures and other biomarkers. CSF CXCL13 levels are correlated with Expanded Disability Status Scale scores, annualized relapse rates (ARR), CSF white blood cells counts, and other measures in NMO and MS (Alvarez et al., 2013; Zhong et al., 2011), and serum CXCL13 levels are correlated with remission extent and magnetic resonance imaging activity during the first and second years, but not with interferon‐β1b or glatiramer acetate levels in MS patients (Festa et al., 2009). To date, most studies regarding CXCL13 have focused on the acute phases of NMO and MS. CXCL13 concentrations during remission remains unclear, and the correlation between serum CXCL13 levels and the use of immunosuppressive therapy (azathioprine, steroids), the first‐line treatment of NMO during remission, has not been studied.

B‐cell‐activating factor, also known as B lymphocyte stimulator or the tumor necrosis factor (TNF) superfamily member 13B (TNFSF13B), is another key cytokine in B‐cell development (Schneider et al., 1999). BAFF levels are increased in systemic lupus erythematosus, rheumatoid arthritis, and Sjögren syndrome, among other conditions (Morais, Vilas‐Boas, & Isenberg, 2015). Elevated CSF BAFF levels are also observed in the acute relapse phase of NMO, which differs from that of MS (Quan et al., 2013; Vaknin‐Dembinsky, Brill, Orpaz, Abramsky, & Karussis, 2010; Wang et al., 2012). Although no differences were seen in serum BAFF levels among NMO patients, MS patients, and healthy donors, increased serum BAFF levels were found in MS patients treated with interferon‐β (Krumbholz et al., 2008; Vaknin‐Dembinsky et al., 2010) and NMO patients treated with rituximab (Gredler et al., 2013). In other words, common therapeutic modalities used to treat NMO and MS therapies affect serum BAFF levels. During remission, most patients with NMO receive treatment consisting of azathioprine and steroids, two nonspecific immunosuppressive agents. We suspected that immunosuppressive therapy could inhibit BAFF expression and that higher serum BAFF levels might exist in NMO patients without treatment during remission, which would indicate persistent humoral immune dysfunction.

Here, we aimed to confirm whether serum CXCL13 and BAFF levels were abnormal in NMO and MS patients during remission and whether we could discriminate between the two diseases accordingly. We also investigated the effect of immunosuppressive therapy on CXCL13 and BAFF expression in NMO.

## 2 Materials and methods

### 2.1 Patients and controls

Twenty‐four NMO patients who met the 2006 Wingerchuk diagnostic criteria (Wingerchuk, Lennon, Pittock, Lucchinetti, & Weinshenker, 2006) and 20 MS patients who met the 2010 McDonald's diagnostic criteria (Polman et al., 2011) from Beijing Tiantan Hospital were enrolled. Serum samples were collected during remission of MS and NMO (>1 month from the last relapse and without new symptoms). Three of the relapsing–remitting MS (RRMS) patients were using Betaseron and the other 17 RRMS patients had no treatments. A total of 16 NMO patients were using immunosuppressive therapy (oral glucocorticoids, *n *=* *7; azathioprine, *n *=* *2; oral glucocorticoids combined with azathioprine, *n *=* *7); Twenty‐two volunteers without CNS inflammatory demyelinating diseases were recruited as controls. The study was approved by the local ethics committee (IRB of Beijing Tiantan Hospital Affiliated to Capital Medical University, No. KY2015‐003‐02) and informed consent was obtained from all participants.

### 2.2 Enzyme‐linked immunosorbent assay

The serum samples were centrifuged with 1220g for 10 min and stored at −80°C. Serum CXCL13 and BAFF levels were measured by a human CXCL13 (Catalog No. DCX130, Lot No. 316603) and BAFF (Catalog No. DBLYS0B, Lot No. 312007) enzyme‐linked immunosorbent assay (Quantikine; R&D Systems, Minneapolis, MN, USA).

### 2.3 Statistical analysis

The data were analyzed using SPSS version 17.0 (SPSS Inc., Chicago, IL, USA. RRID: SCR_002865). The different groups were compared with nonparametric Mann–Whitney *U* test or Student's *t* test according to normality. Correlations between CXCL13/BAFF levels and clinical features (onset age, annualized relapse rate, disease course, and time from the last relapse) were analyzed using Spearman's rank test.

### 2.4 Compliance with ethical standards

This study was approved by the local ethics committee (IRB of Beijing Tiantan Hospital Affiliated to Capital Medical University, No. KY2015‐003‐02) and informed consent was obtained from all participants.

## 3 Results

### 3.1 Patient demographics

The mean ages of the groups (NMO, MS, and control) were similar. The demographics and clinical features of NMO and MS patients are shown in Table 1 and details in Table 2.

**Table 1:** Participants demographics

	NMO	MS	Control
Number	24	20	22
Gender, female/male	22/2	14/6	16/6
Age, mean	34.63	31.80	33.59
Onset age, median (range)	28 (13–53)	25 (6–60)	–
Relapse frequency, median (range)	4 (1–8)	3 (1–10)	–
Disease duration, median (range)	43.50 (2–258)	29 (2–143)	–
Annualized relapse rate, median (range)	0.80 (0–3.20)	0.65 (0–2.86)	–
Duration to the last relapse, median (range)	4 (1.5–33)	4 (1.5–40)	–

NMO, neuromyelitis optica; MS, multiple sclerosis.

Age refers to age of visiting time.

–, not available.

**Table 2:** Demographic and clinical data of NMO

Pt No.	Age (year)/gender	Disease dur (month)	Dur to the last relapse	EDSS	No. of relapse	ImmoS therapy
NMO‐1	24/F	114	33	6	7	−
NMO‐2	20/F	55	15	3	4	+
NMO‐3	55/F	30	4	3.5	3	−
NMO‐4	22/F	38	4	1.5	4	+
NMO‐5	14/F	18	2	2	5	+
NMO‐6	51/F	43	6	5.5	6	+
NMO‐7	39/F	15	11	2	2	−
NMO‐8	42/F	6	2	2	2	+
NMO‐9	46/F	108	1.5	3.5	3	+
NMO‐10	26/F	40	9	2.5	6	+
NMO‐11	34/M	15	5	1.5	5	+
NMO‐12	31/F	82	5	2.5	8	−
NMO‐13	43/F	107	3	3	3	+
NMO‐14	37/F	71	9	1	6	+
NMO‐15	29/F	14	6	1.5	3	+
NMO‐16	39/F	122	3	3.5	4	+
NMO‐17	34/F	44	1.5	1	4	+
NMO‐18	53/F	258	10	3.5	2	+
NMO‐19	40/M	2	2	2	1	−
NMO‐20	31/F	2	1	3.5	1	+
NMO‐21	36/F	150	1	5.5	6	−
NMO‐22	32/F	81	1	2	5	−
NMO‐23	19/F	30	1	3	4	−
NMO‐24	32/F	73	1	6	4	+

Pt, patients; No, number; Dur, duration; ImmoS, immunosuppressive; F, female; M, male.

### 3.2 Serum CXCL13 levels

**Figure 1:**
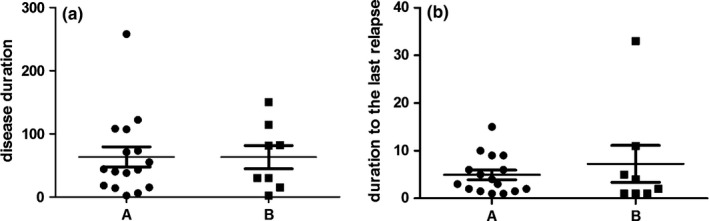
Disease duration and duration to the last relapse in NMO patients. (a) Disease duration in patients with NMO treated with immunosuppressive agents (group A, *n *=* *16), patients with NMO untreated with immunosuppressive agents (group B, *n *=* *8). (b) Duration to the last relapse in patients with NMO treated with immunosuppressive agents (group A, *n *=* *16), patients with NMO untreated with immunosuppressive agents (group B, *n *=* *8)

Compared with the control group (median, 75.16 pg/ml; range, 27.70–279.71), the serum CXCL13 levels were higher in patients with NMO (median, 156.32 pg/ml; range, 46.88–398.22) (Z = −3.298, *p *=* *.001), and they also had a higher trend than those of MS patients (median, 90.52 pg/ml; range, 36.50–530.94) (Z = −1.650, *p *=* *.099). Serum CXCL13 levels in MS patients were not significantly higher than those in the control group (Z = 1.083, *p *=* *.279) (Figure 1b).

### 3.3 Disease duration and duration to the last relapse in NMO

There were no significant differences in terms of disease duration and duration to the last relapse between NMO patients treated with immunosuppressive agents (group A, *n *=* *16) and NMO patients who were not treated with immunosuppressive agents (group B, *n *=* *8) (Figure 1b).

### 3.4 CXCL13 and immunosuppressive therapy in NMO

**Figure 2:**
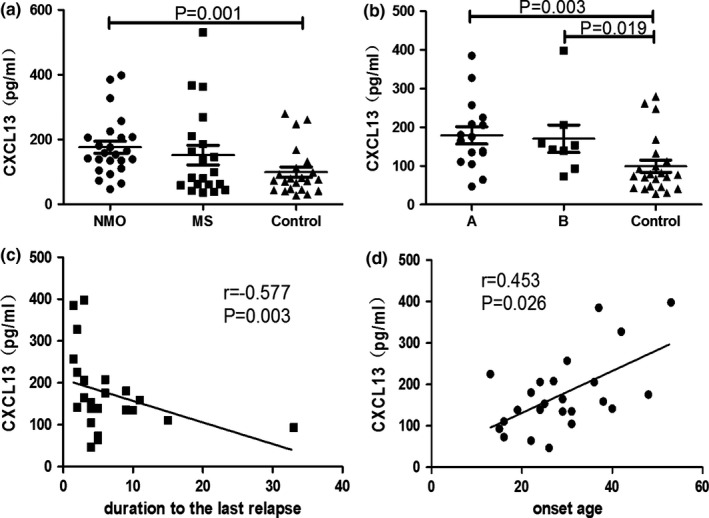
Serum CXCL13 levels. (a) Serum CXCL13 levels of neuromyelitis optica (NMO), multiple sclerosis (MS), and control group (mean ± SE). (b) Serum CXCL13 levels in patients with NMO treated with immunosuppressive agents (group A, *n *=* *16), patients with NMO untreated with immunosuppressive agents (group B, *n *=* *8), and controls (control group) (mean ± SE). (c, d) Correlation between CXCL13 and duration to the last relapse or the onset age

There were no significant differences between serum CXCL13 levels in the 16 NMO patients using immunosuppressive agents (median, 170.28 pg/ml; range, 46.88–385.39) and the other eight NMO patients (median, 147.73 pg/ml; range, 73.10–398.22) (Figure 2a,b).

### 3.5 CXCL13 correlation with clinical features in NMO

In NMO patients, CXCL13 was correlated with onset age (r = .453, *p *=* *.026) (Figure 2c) and duration to the last relapse (in months) (r = −.577, *p *=* *.003) (Figure 2d), but not with relapse frequency (r = −.161, *p *=* *.454), disease duration (r = −.055, *p *=* *.798), or ARR (r = .126, *p *=* *.558).

### 3.6 Serum BAFF levels

Median serum BAFF levels in the NMO, MS, and control groups were 945.52 pg/ml (range, 278.14–1,942.81), 940.05 pg/ml (range, 245.60–1,722.99), and 962.40 pg/ml (range, 779.04–1,333.87). There were no significant differences among the three groups (NMO vs MS, *t *=* *−0.321, *p *=* *.749; NMO vs control, Z = −0.572, *p *=* *.567; MS vs control, Z = −0.126, *p *=* *.900) (Figure 2a).

### 3.7 BAFF and immunosuppressive therapy in NMO

**Figure 3:**
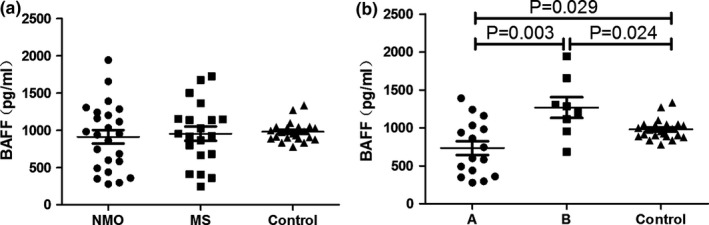
Serum BAFF levels. (a) Serum BAFF levels of neuromyelitis optica (NMO), multiple sclerosis (MS), and control group (mean ± SE). (b) Serum BAFF levels in patients with NMO treated with immunosuppressive agents (group A, *n *=* *16), patients with NMO untreated with immunosuppressive agents (group B, *n *=* *8), and controls (control group) (mean ± SE)

Median serum BAFF levels in the 16 NMO patients using immunosuppressive agents (671.66 pg/ml; range, 278.14–1,389.76) was lower than those of the other eight NMO patients (1,243.36 pg/ml; range, 685.09–1,942.81) (*t *=* *−3.325, *p *=* *.003) and the controls (Z = −2.188, *p *=* *.029). Serum BAFF levels in the other eight NMO patients were much higher than those of the control group (Z = −2.251, *p *=* *.024) (Figure 3b).

### 3.8 BAFF correlation with clinical features and CXCL13 in NMO

In NMO patients, BAFF levels were not correlated with onset age (r = .328, *p *=* *.118), relapse frequency (r = .163, *p *=* *.447), disease duration (r = .125, *p *=* *.561), ARR (r = −.202, *p *=* *.343), or duration to the last relapse (in months) (r = .267, *p *=* *.208). There was also no significant correlation between CXCL13 and BAFF levels (r = −.104, *p *=* *.628).

## 4 Discussion

Here, we analyzed serum CXCL13 and BAFF levels in NMO and MS patients during remission and explored correlations of CXCL13 and BAFF levels with the clinical features and immunosuppressive treatment of NMO patients. First, we confirmed that serum CXCL13 levels were elevated during remission and that serum BAFF levels of patients with NMO that were untreated with immunosuppressive agents were higher than those of controls.

A number of studies have shown that NMO‐IgG, a specific anti‐AQP4 autoantibody produced in the humoral immune system by B cells, can be detected in most patients with NMO (Jarius & Wildemann, 2010) and is pathogenic. In addition, B‐cell depletion therapy with rituximab is effective against NMO (Collongues et al., 2015; Zephir et al., 2015). Accordingly, B cells definitely have an important role in NMO pathogenesis. One study reported that serum NMO‐IgG titers were 500 times higher than those in the CSF, which demonstrates that NMO‐IgG is produced in the peripheral blood but not in the intrathecal area (Takahashi et al., 2007). Research to date has indicated that B‐cell‐mediated inflammation exists not only in the CNS but also in the peripheral blood of NMO patients. Therefore, peripheral B‐cell‐mediated inflammation is correlated with disease activity in NMO. In this study, we detected crucial peripheral factors involved in B‐cell‐mediated inflammatory reaction. CXCL13 interacts with CXCR5 on B cells and promotes B‐cell development within the secondary lymphoid tissues by chemotactic recruitment (Legler et al., 1998). BAFF is a major factor for peripheral immature B lymphocyte survival and differentiation in response to signals via the B‐cell receptor (Batten et al., 2000). Both play important roles in B‐cell‐mediated inflammation.

In the relapse phase, CD138^+^HLA^−^DR^+^ plasma blasts are increased in the peripheral blood of patients with NMO (Chihara et al., 2013) and cause severe inflammatory activity. The serum gradients of CXCL13 are higher in NMO patients within 2 months of symptom relapse than in noninflammatory neurological disease controls (Alvarez et al., 2013). Significantly higher levels of BAFF in the CSF of NMO patients have been reported compared with MS patients and healthy donors (Vaknin‐Dembinsky et al., 2010). There is evidence that inflammation exists in all stages of MS (Frischer et al., 2009), but studies have seldom focused on the remission period of NMO.

Our research focused on NMO patients during remission and resulted in a few main findings. First, we found significantly increased CXCL13 levels in NMO, a finding that is consistent with previous studies of the relapse phase (Alvarez et al., 2013; Quan et al., 2013). This result suggested that B‐cell‐mediated inflammation persisted even in the remission stage. No difference in serum BAFF levels was observed between NMO patients and controls; however, obviously higher BAFF levels were noted in NMO patients after the exclusion of treatment factors, although only eight patients did not receive treatment. This result further illustrates that the disease was not absolutely stable and that humoral immune dysfunction persisted during NMO remission.

There were no significant differences in CXCL13 and BAFF levels between NMO and MS patients. Although there was a higher trend of CXCL13 and BAFF levels in NMO vs MS, both diseases exhibited a wide range of levels. Thus, we could not distinguish between the two diseases by these levels alone. B cells contribute to the pathogenesis of both NMO and MS, in which CXCL13 and BAFF play roles (Krumbholz & Meinl, 2014). According to previous research, we cannot discriminate between the two diseases by CSF and serum CXCL13 concentrations, even in the acute phase (Alvarez et al., 2013).

This study elucidated the effect of immunosuppressive therapy on BAFF levels. NMO patients with immunosuppressive treatment (azathioprine, steroids) had significantly lower BAFF levels than the other NMO patients and the controls. These findings indicate that immunosuppressive treatment might delay relapse in NMO by decreasing serum BAFF concentrations. Due to the lack of MS patients with immunosuppressive treatment, this study was not able to conclude whether the effect of immunosuppressive treatment on BAFF is specific to NMO patients.

Azathioprine is a purine synthesis inhibitor that interferes with cellular proliferation, particularly leukocytes. Azathioprine in combination with oral prednisolone has been shown to be an effective treatment for NMO and has been recommended as the first‐line treatment during remission (Sellner et al., 2010). Our study revealed that BAFF was one of its targets and confirmed the necessity of use in NMO remission. There were no significant differences between serum CXCL13 levels in NMO patients with immunosuppressive therapy and other NMO patients. CXCL13 is secreted in the secondary lymphoid organs by follicular dendritic cell and macrophages (Carlsen et al., 2004; Gunn et al., 1998). We suspect that immunosuppressive treatment had little influence on these cells and could not affect CXCL13 expression. One murine model study showed that anti‐CXCL13 antibody was effective in the treatment of autoimmune disorders (Klimatcheva et al., 2015). Despite other studies that have demonstrated that CXCL13 is elevated in many inflammatory CNS diseases and is not specific to NMO and MS, our findings show that therapy targeting CXCL13 may also be useful for NMO (Kothur, Wienholt, Brilot, & Dale, 2016).

Finally, we discovered that serum CXCL13 levels declined with prolonged time to the last relapse, which suggests that it is correlated with disease activity. High concentrations of CXCL13 were unfavorable for NMO recovery. However, immunosuppressive treatment was ineffective for CXCL13 expression. Accordingly, we suspect that therapy targeting CXCL13 in combination with immunosuppressive treatment may be more useful in reducing NMO recurrence. Furthermore, this study showed that serum CXCL13 levels were positively correlated with NMO onset age. This may mean that NMO patients with an older onset age may have a more severe extent of inflammation.

## 5 Conclusion

In summary, serum levels of both CXCL13 and BAFF in NMO patients remained higher during remission, which shows that humoral immune dysfunction persisted during NMO remission. Furthermore, immunosuppressive therapy can decrease serum BAFF levels but only slightly affects CXCL13 levels. As such, we suspect that immunosuppressive treatment is necessary and useful and that CXCL13 targeting may be another effective therapy for reducing NMO recurrence. This study was limited in patients who were not examined before and after treatment and during remission, and this may be a potential reason that significant differences in BAFF levels were not observed between NMO patients with and without treatment.

## Author contributions

Su Wang, Tao Yang, and Jianglong Wan participated in the collection of samples and carried out CXCL13 and BAFF ELISA tests. Su Wang and Yongchao Zhang performed the statistical analysis. Yongping Fan was the designer of the study and took overall clinical and research responsibility. All authors provided their final approval of the content of this manuscript.

## Conflicts of Interest

The authors declare no conflicts of interest.
